# The MACADAM database: a MetAboliC pAthways DAtabase for Microbial taxonomic groups for mining potential metabolic capacities of archaeal and bacterial taxonomic groups

**DOI:** 10.1093/database/baz049

**Published:** 2019-04-29

**Authors:** Malo Le Boulch, Patrice Déhais, Sylvie Combes, Géraldine Pascal

**Affiliations:** 1 GenPhySE, Université de Toulouse, INRA, INPT, ENVT, Castanet Tolosan, France; 2 Sigenae Group, GenPhySE, Université de Toulouse, INRA, INPT, ENVT, Castanet Tolosan, France

## Abstract

Progress in genome sequencing and bioinformatics opens up new possibilities, including that of correlating genome annotations with functional information such as metabolic pathways. Thanks to the development of functional annotation databases, scientists are able to link genome annotations with functional annotations. We present MetAboliC pAthways DAtabase for Microbial taxonomic groups (MACADAM) here, a user-friendly database that makes it possible to find presence/absence/completeness statistics for metabolic pathways at a given microbial taxonomic position. For each prokaryotic ‘RefSeq complete genome’, MACADAM builds a pathway genome database (PGDB) using Pathway Tools software based on MetaCyc data that includes metabolic pathways as well as associated metabolites, reactions and enzymes. To ensure the highest quality of the genome functional annotation data, MACADAM also contains MicroCyc, a manually curated collection of PGDBs; Functional Annotation of Prokaryotic Taxa (FAPROTAX), a manually curated functional annotation database; and the IJSEM phenotypic database. The MACADAM database contains 13 509 PGDBs (13 195 bacterial and 314 archaeal), 1260 unique metabolic pathways, completed with 82 functional annotations from FAPROTAX and 16 from the IJSEM phenotypic database. MACADAM contains a total of 7921 metabolites, 592 enzymatic reactions, 2134 EC numbers and 7440 enzymes. MACADAM can be queried at any rank of the NCBI taxonomy (from phyla to species). It provides the possibility to explore functional information completed with metabolites, enzymes, enzymatic reactions and EC numbers. MACADAM returns a tabulated file containing a list of pathways with two scores (pathway score and pathway frequency score) that are present in the queried taxa. The file also contains the names of the organisms in which the pathways are found and the metabolic hierarchy associated with the pathways. Finally, MACADAM can be downloaded as a single file and queried with SQLite or python command lines or explored through a web interface.

## Introduction

For many years, *Bergey’s Manual of Determinative Bacteriology* (1) and its successor, *Bergey’s Manual of Systematic Bacteriology* (2–6), which provides descriptions of the taxonomy, systematics, ecology, physiology and other biological properties of all described prokaryotic taxa, has been the best consensus for an official prokaryotic classification and the best source of information for prokaryotic organisms and taxa. Thanks to advances in genome sequencing and bioinformatics, it is now possible to link genome annotations and functional information. To make this possible, databases have been built and contain metabolic pathways, e.g. series of chemical reactions catalyzed by enzymes within a cell. For instance, the KEGG database (7) can display any of these pathways in a graphical environment. The Human Metabolome DataBase (HMDB) (8) and Reactome (9) are highly curated and complete databases specializing in human metabolism. WikiPathways (10) is an open access collaborative platform containing metabolic pathways across different species. PATRIC (11) is a bacterial database containing >201 000 prokaryotic genomes, each associated with functional information. BioCyc (12) links the genome sequence of an organism to its functional annotation in >14 560 eukaryotic, bacteria and archaea species. All these databases are referred to as pathway genome databases (PGDBs); i.e. they associate the genome sequences with metabolic pathways. Currently, among available databases, some databases are highly curated, including the EcoCyc (13), BsubCyc (14) and HumanCyc (15) databases devoted to *Escherichia coli* K-12, *Bacillus subtilis* or human metabolic pathways, respectively. They are based on the MetaCyc (16) database, which is a highly curated database containing >2666 metabolic pathways throughout the living world. The MicroCyc database (17), based on the MetaCyc database, has been improved by automatic and manual curation by specialized biologists. Finally, some other databases are also curated using functional information from the literature, e.g. FAPROTAX (18) or the IJSEM phenotypic database (19, 20).

Each of these latter databases has limits to link microbial taxonomy to functional information and is not easily downloadable. HMDB, Reactome and WikiPathways are manually and highly curated, but, despite the high quality of their functional information, they cover a small number of organisms [1, 1 and 31 (3 of which are microbes), respectively]. KEGG, despite having >5299 prokaryotic organisms, has moved to a subscription mode and cannot be accessed offline. PATRIC depends on the KEGG pathway data and cannot be downloaded (Table 1). The BioCyc database is a microbial genome web portal that combines thousands of genomes with pathway information, but the BioCyc website uses a subscription model, free access to the derived BioCyc database is limited to a 2-year-old collection of PGDBs and online consultation is limited to a specific number of times per month. Further access requires a paid subscription. MetaCyc contains a greater number of metabolic pathways than KEGG (21) (2666 vs. 530 metabolic pathways, respectively), and it is freely available for academics, but only a few metabolic pathways are retrievable via a taxonomy or an organism name. With existing databases, it is difficult to request up-to-date functional annotations and up-to-date taxonomic lineages for a taxon (e.g. a whole family). But one MACADAM feature is not provided by these databases: the possibility to infer functional information for prokaryotic taxa with no functional information associated with it. That is why we built MACADAM (MetAboliC pAthways DAtabase for Microbial taxonomic groups), a user-friendly database that makes it possible to find presence/absence/completeness statistics for metabolic pathways at a given archaeal and bacterial taxonomic rank or organism and to be able to infer functional information using the taxonomy for taxa without functional information. MACADAM is not intended to replace existing databases but provides additional information for scientists wishing to better characterize the functional information of all taxonomic groups, from phyla to species.

**Table 1:** Overview of MACADAM, BioCyc, PATRIC and KEGG database features with a focus on metabolic pathway and functional information among prokaryotic organisms

	**MACADAM**	**BioCyc**	**PATRIC**	**KEGG**
**Microbial taxonomy used for requests**	NCBI taxonomy	NCBI taxonomy	NCBI taxonomy	KEGG taxonomy (with cross-link to NCBI)
**Query possibilities**	On one or several taxonomies or organisms, with few filters	On one organism, with multiple filters	On one or several taxonomies or organisms, with multiple filters	On one organism, with no filters
**Number of bacterial organisms**	13 195	~13 400	198 855	5014
**Number of archaeal organisms**	314	~400	3069	285
**Number of unique metabolic pathways**	1260	2666	143	530
**Genome origins**	RefSeq (complete genomes)	Genbank and RefSeq	Genbank and RefSeq	Genbank and RefSeq
**Functional annotation sources**	RefSeq (functional annotations), MetaCyc (metabolic pathways), MicroCyc (metabolic pathways), FAPROTAX (functional features), IJSME PhenoDB (phenotypic data)	Genbank/RefSeq (annotations) and MetaCyc (metabolic pathways)	Genbank/RefSeq, KEGG (metabolic pathways)	KEGG
**Analysis tools and metrics**	PS and PFS	SmartTables, genome browser, omics data analysis, metabolic models and routes and comparative analysis	KEGG pathway map, comparative pathway heatmap, multiple sequence alignment, enzymes and genes conservation in pathway	KEGG mapper tools
**Output data types**	Metabolic pathway name, pathway class hierarchy, hyperlink to MetaCyc pathway and functional information of the upper rank for taxa without data	Metabolic pathway name, pathway class hierarchy, pathway map including enzymes and metabolites, associated genes, protein associated with pathways and literature references	Metabolic pathway name, pathway class hierarchy, KEGG pathway map including enzymes and metabolites, associated genes, enzyme and gene evolution data	Metabolic pathway name, pathway class hierarchy, KEGG pathway map including enzymes and metabolites, associated genes and literature references
**Database flat files downloadable**	Yes	Yes for academics	No	Yes with license
**Results downloadable**	Yes	Yes	Yes	Available on the web only
**Command line interrogation**	SQLite or python script	Application Programming Interface (API)	API + free command line software	API
**Frequency of updates**	6 months	2–6 months	6 months	3 months

**Figure 1:**
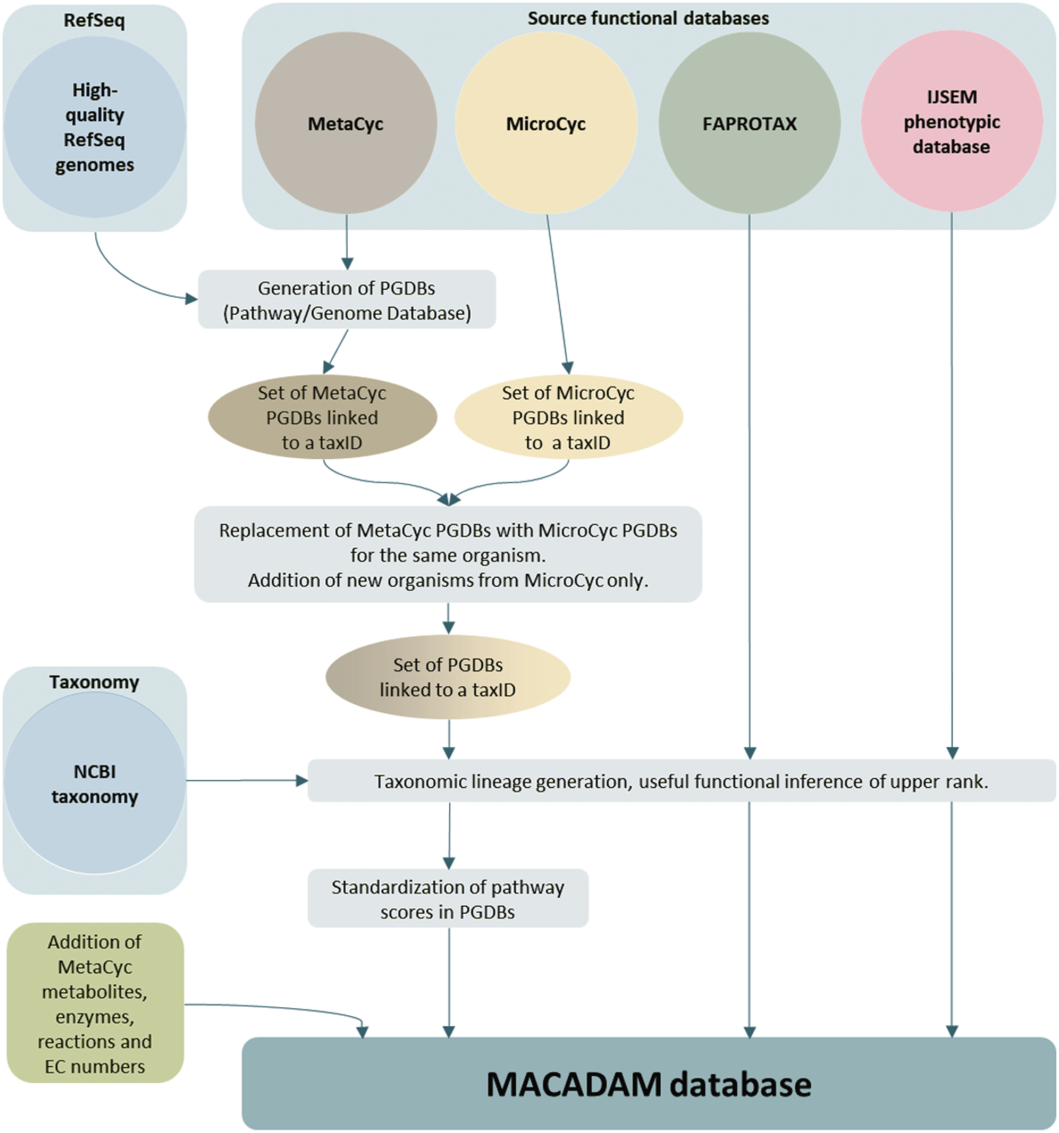
MACADAM building workflow.

## MACADAM: main characteristics and added value

The following is an introduction to the MACADAM database’s main characteristics and improvements compared to existing databases. Its advantages are 4-fold. First, the user has the possibility to explore functional traits at different taxonomic levels, from phyla to species, or dedicated strains covering both the *Archaea* and *Bacteria* kingdoms. The user can request data about several taxonomies or organisms at the same time and can do it using partial names. Second, MACADAM relies on up-to-date NCBI taxonomy (22–24). Third, it is based on high-quality genome functional annotations thanks to the aggregation of (i) up-to-date RefSeq genomes annotated with MetaCyc functional data, (ii) high-quality functional annotations from the MicroCyc database (25) and (iii) the FAPROTAX and IJSEM phenotypic databases (manually curated databases). Fourth, MACADAM is open access, freely downloadable and the possibilities of consultation are unlimited. Altogether, MACADAM facilitates taxonomical functional inference studies by synthesizing high-quality and up-to-date functional annotations and taxonomical information in one source. Table 1 shows the comparison between MACADAM, BioCyc, PATRIC and KEGG databases in the context of queries on functional and metabolic pathway information for microbial organisms. WikiPathways does not appear in Table 1 because it contains only three microbial organisms and it is not useful for microbial queries.

## MACADAM content

### Building PGDBs between RefSeq and MetaCyc

To build the MACADAM database (Figure 1), we first sourced genomic data of prokaryotes from NCBI RefSeq (25), version 92 (16 January 2019). To ensure high-quality genomic data, we only kept the 11 794 organisms (11 514 bacteria and 280 archaea) with ‘complete genome’ RefSeq labels (Table 2). These genomes have no gaps, no runs of 10 or more ambiguous bases and the entire chromosome is present (26). This quality allows better annotations using the NCBI prokaryotic genome annotation pipeline (27). Out of the 11 794 genomes, 1683 were labeled ‘representative genomes’ (1543 bacteria and 140 archaea), i.e. a subset of genomes with an additional quality assurance analysis based on annotation quality metrics. In addition, 118 bacteria are labeled ‘reference genome’ (no archaea). They correspond to the highest quality data set, supported by the curation of NCBI scientific staff, and are manually curated. To associate metabolic pathway information with these genomes, we used Pathway Tools software (28) (version 20.5) that relies on MetaCyc data. We thus build a PGDB per organism, i.e. 11 794, that consists of a list of pathways based on the genome annotation. Moreover, to complete pathway information, MACADAM includes the metabolites, reactions, EC numbers, enzymes and hierarchical classification of each pathway. The result is a set of PGDBs and an up-to-date functional annotation of multiple organisms.

**Table 2:** Statistics on PGDBs collected for MACADAM (values are mean ± SD; values in brackets are minimum and maximum values)

	**Number of organisms**	**Pathways per organism**	**Number of different pathways**	**PS**	**PFS**
MetaCyc PGDBs	9954	156 ± 60 [3–350]	851	0.85 ± 0.21 [0–1]	1.37 ± 1.21 [0–51]
MicroCyc PGDBs that have replaced a MetaCyc PGDB	1560	247 ± 80 [26–425]	1012	0.75 ± 0.28 [0.344–1]	1.37 ± 1.45 [0.344–77]
PGDBs only present in MicroCyc	1681	255 ± 70 [2–422]	1007	0.75 ± 0.28 [0.344–1]	1.32 ± 1.28 [0.344–47]
**MACADAM bacteria**	**13 195**	**179 ± 76 [2–425]**	**1224**	**0.82 ± 0.24 [0–1]**	**1.36 ± 1.26 [0–77]**
MetaCyc PGDBs	184	60 ± 14 [16–91]	207	0.84 ± 0.22 [0.2–1]	1.35 ± 1.11 [0.2–15]
MicroCyc PGDBs that have replaced a MetaCyc PGDB	96	107 ± 25 [2–156]	393	0.77 ± 0.28 [0.05–1]	1.28 ± 1.12 [0.05–21]
PGDBs only present in MicroCyc	34	98 ± 22 [8–149]	344	0.77 ± 0.28 [0.05–1]	1.21 ± 0.93 [0.05–16]
**MACADAM archaea**	**314**	**79 ± 29 [2–156]**	**478**	**0.80 ± 0.26 [0.05–1]**	**1.31 ± 1.10 [0.05–21]**
**MACADAM total**	**13 509**	**177 ± 76 [1–425]**	**1260**	**0.82 ± 0.24 [0–1]**	**1.36 ± 1.26 [0–77]**

in bold: summary of metrics for bacteria, archaea or both.

### Embedding PGDBs from MicroCyc

**Figure 2:**
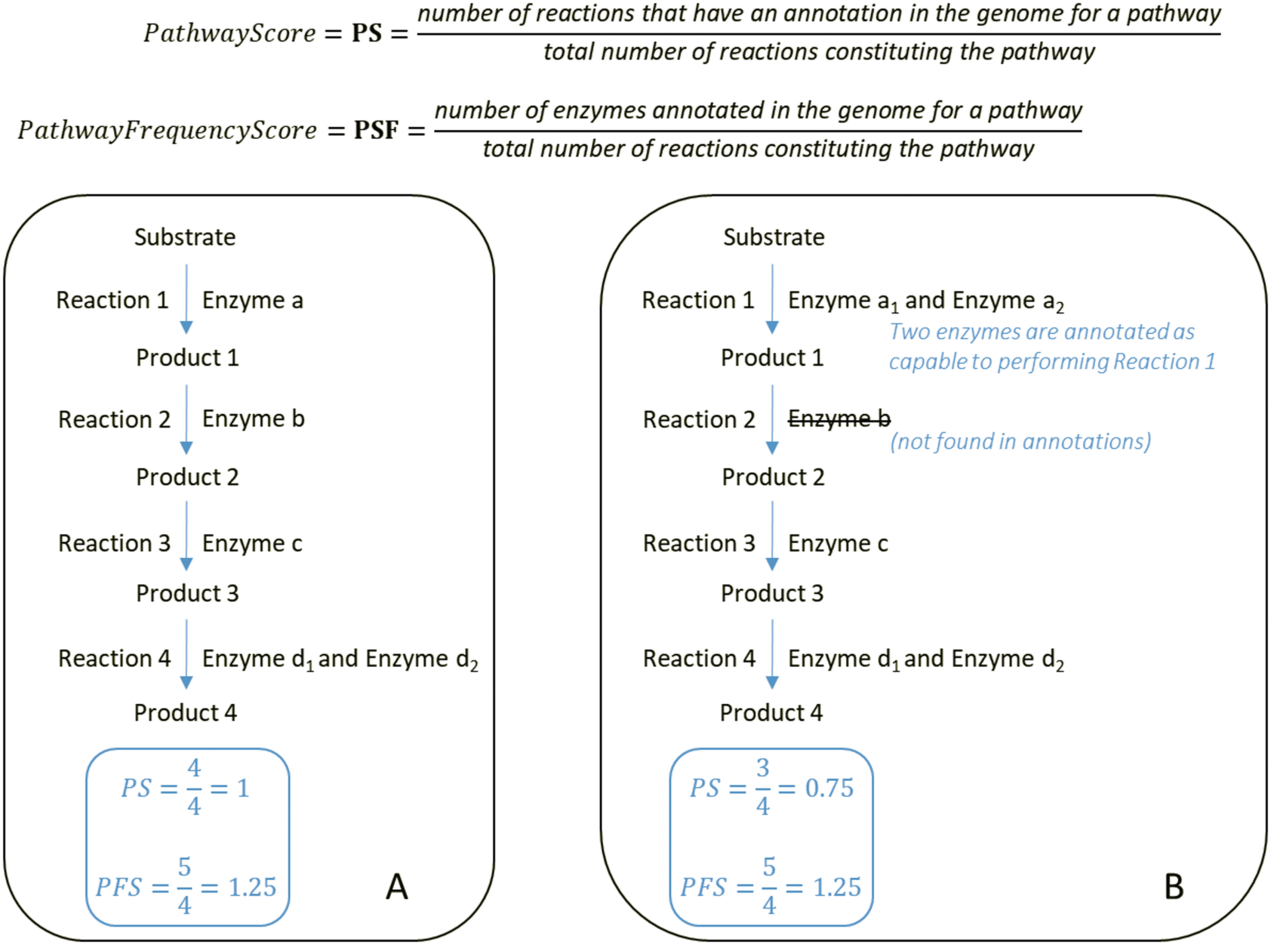
At the top, formulas for the computation of the PS and PFS. Below, examples of two types of computations of the PS and PFS based on an example of a hypothetical metabolic pathway. By comparing the PS and PFS in **A** and **B**, pathway A shows greater evidence of its veracity.

**Figure 3:**
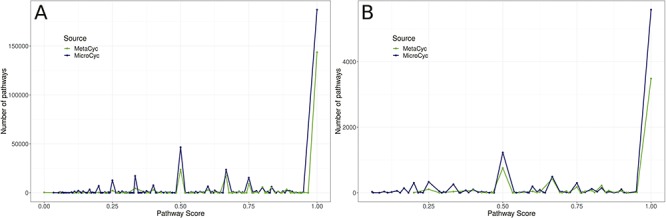
Comparison of PS of all metabolic pathways of PGDBs present in both MetaCyc PGDBs and MicroCyc PGDBs. **A**: among *Bacteria*; **B**: among *Archaea*.

In the second step of MACADAM building, we added data from MicroCyc (17). MicroCyc is a collection of PGDBs created within the framework of the MicroScope project (29) based on MetaCyc data. These PGDBs are generated from genomes that are (i) manually (re)annoted, (ii) improved thanks to the addition of enzymatic function predictions that are computed with PRIAM software (30) and (iii) completed by annotations from biologist curations using the MaGe system (31). Using the standardized pathway score (PS; Figure 2) proposed in Pathway Tools 16.5, we compare MetaCyc and MicroCyc PGDBs for each organism that is present in both databases. As expected, due to the curation process, MicroCyc PGDBs exhibit more metabolic pathways with a PS equal to 1 than MetaCyc PGDBs (Figure 3). Moreover, MicroCyc contains more pathways per organism than MetaCyc (Table 1). Thus, for common PGDBs between the two databases, those from MicroCyc are chosen over those from MetaCyc and are included in MACADAM, i.e. a total of 1656 PGDBs (1560 bacteria and 96 archaea). MACADAM is then enriched with MicroCyc PGDBs of organisms that are absent from MetaCyc PGDBs, i.e. 1715 (1681 bacteria and 34 archaea). To be added to MACADAM, MicroCyc PGDBs have to be linked to a prokaryotic organism and have a taxonomy recognized in the NCBI taxonomy. As for the MetaCyc process, MACADAM includes the metabolites, reactions, EC numbers, enzymes and hierarchical classification of each pathway. We therefore obtain an improved set of PGDBs from MetaCyc and MicroCyc, completed with functional annotations of 13 509 organisms (Table 1).

### Embedding functional annotations from the FAPROTAX and IJSEM phenotypic database

In the third step of MACADAM construction, we added functional traits extracted from the FAPROTAX database (18) and the IJSEM phenotypic database (19). FAPROTAX contains soil and marine bacteria. FAPROTAX maps prokaryotic clades (e.g. genera or species) to establish metabolic or other ecologically relevant functions using the current literature on cultured strains, e.g. *Bergey’s Manual of Systematic Bacteriology* (2–6), the Prokaryotes (32) and the IJSEM journal (33). The IJSEM phenotypic database contains phenotypic, metabolic and environmental tolerance data of prokaryotic strains manually extracted from articles in the IJSEM journal. We completed MACADAM with 82 functional annotations from FAPROTAX and 16 from the IJSEM phenotypic database (Table 3). Since this information has a different structure, the PS cannot be calculated.

### Taxonomy in MACADAM

To link this information, we connected data from PGDBs, the FAPROTAX and the IJSEM phenotypic database with their NCBI taxonomy ID (taxID). This taxID is a unique time-stable identifier because of its interoperability with other taxonomic databases (34). Briefly, in MACADAM, each organism taxonomy has seven taxonomic ranks: superkingdom, phylum, class, order, family, genus and species. Each rank and organism is described by its NCBI taxID. Each MACADAM organism is therefore associated with its numeric lineage formed by seven taxID. For example, *E. coli* is described as *Bacteria*, *Proteobacteria*, *Gammaproteobacteria*, *Enterobacterales*, *Enterobacteriaceae*, and *Escherichia*. *E. coli* is linked to this lineage 2.1224.1236.91347.543.561.562 in MACADAM. However, even if MACADAM data are not associated with minor ranks, e.g. subclasses or subgenera, these minor ranks are kept in MACADAM so that users can find functional information at these minor ranks.

In conclusion, to create MACADAM, we need (i) RefSeq ‘complete genomes’, (ii) Pathway Tools with MetaCyc, (iii) MicroCyc PGDBs, (iv) NCBI taxonomy files, (v) the FAPROTAX file and (vi) the IJSEM phenotypic database file.

## MACADAM: structure and management

### Structure of the MACADAM database

**Figure 4:**
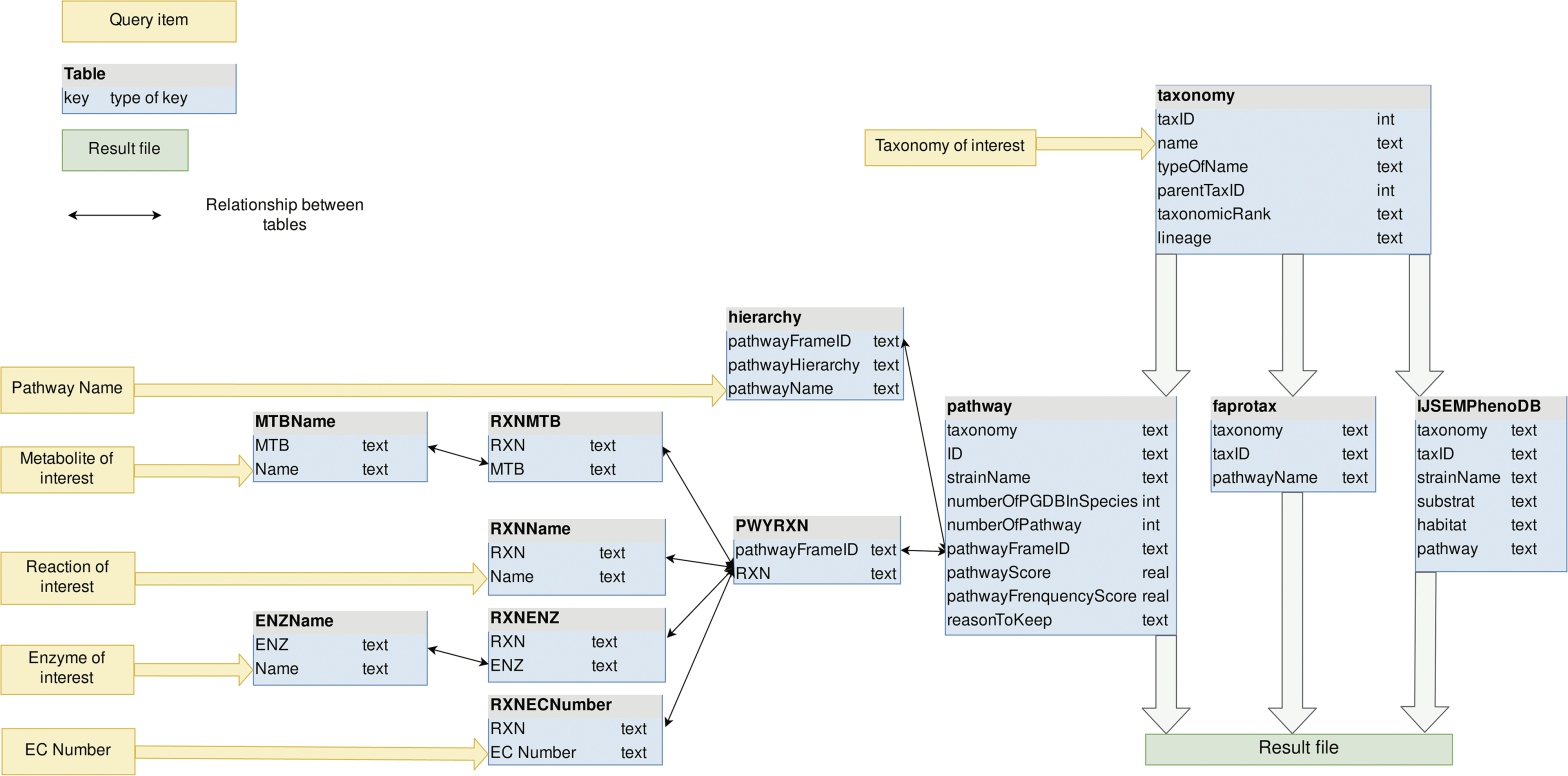
MACADAM database schema. Yellow arrows indicate the entry points of the database.

The MACADAM database is a SQLite database (35) that optimizes and facilitates querying. SQLite is an object-relational database management system. It increases the query speed of databases. MACADAM is organized into 12 tables (Figure 4).

**Table 3:** Statistics on FAPROTAX and IJSEM phenotypic database information in the MACADAM database for bacterial and archaea organisms

	**Number of organisms or taxa**	**Phenotypic, metabolic or environmental tolerance data**
FAPROTAX	*Bacteria*: 3838; *Archaea*: 181	*Bacteria*: 82; *Archaea*: 44
IJSEM phenotypic database	*Bacteria*: 4240; *Archaea*: 87	*Bacteria*: 16; *Archaea*: 12

As shown in Figure 4, the taxonomy table contains all of the prokaryotic taxonomic names of NCBI taxonomy, whether a PGDB exists or not. Indeed, each PGDB is associated with an organism, i.e. with a species or a strain taxID and PGDBs do not exist at upper taxonomic ranks as genus, family and others. However, to provide users with functional information at these upper ranks, MACADAM needs to store all prokaryotic taxonomies. Thus, the corresponding table in MACADAM contains the taxID of each taxonomic name, the taxID of its parent (parentTaxID) and its taxonomic rank name (taxonomicRank), if any, and the numeric lineage up to this taxID, e.g. taxonomic name = *Proteobacteria*—taxID = 1224, parentTaxID = 2, taxonomicRank = phylum—lineage = 2.1224.

An important table is the pathway table (Figure 4). This table is composed of nine keys. The first one is the taxonomy key that is the numeric lineage. The second one is the ID key that is a unique identifier associated with each PGDB. It consists of the taxID of the corresponding organism, the RefSeq genome label (‘rep’ for representative genomes, ‘ref’ for reference genome or ‘nan’ for complete genomes) and its database of origin (MetaCyc or MicroCyc). The third key is the strainName of a true strain name if it exists. Otherwise, it is provided with the species name. The fourth key is the numberOfPGDBInSpecies. For example, MACADAM has 667 PGDBs linked to *E. coli* species. This means that these 667 PGDBs are linked to 667 strains of *E. coli*; thus, the numberOfPGDBInSpecies for *E. coli* is equal to 667. The fifth key is the number of pathways in a PGDB (numberOfpathway). The sixth key is the pathwayFrameID key that is the official unique MetaCyc identifier of each pathway. The seventh and eighth keys are PS and the pathway frequency score (PFS), as described in Figure 2. The last key is the ReasonToKeep key that explains why we decided to keep the pathway in the database. The reasons are either that the pathway is from MicroCyc, that the pathway has a high enough threshold quality score, or that the metabolic pathway is complete.

In the hierarchy table, the pathwayHierarchy corresponds to the pathway functional hierarchy found in MetaCyc. For example, for the nitrogen fixation I (pathwayName) pathway, its hierarchy is Degradation/Utilization/Assimilation—Inorganic Nutrient Metabolism—Nitrogen Compound Metabolism—Nitrogen fixation, and its pathwayFrameID key is N2FIX-PWY. The PWYRXN table binds each pathwayFrameID to all MetaCyc official identifiers of reactions that compose the pathways. Each reaction can be described by its name, its metabolites, its enzymes and its EC numbers. These data are stored in RXNName, RXNMTB, RXNENZ and RXNECNumber tables, respectively. For example, the adenosine deoxyribonucleotide *de novo* biosynthesis pathway is composed, among other things, of ADPREDUCT-RXN, known as ADP reductase (EC number = 1.17.4.1), which reduces the ADP metabolite thanks to the ribonucleoside-diphosphate reductase enzyme.

For each organism encoded with its taxID key, the FAPROTAX table and the IJSEMPhenoDB table contain its numeric lineage (taxonomy key) and complementary information such as functional features (pathwayName or pathway keys), environmental habitat or substrate to culture the organism.

### Management of the MACADAM database update

**How**: The MACADAM database is built from a pipeline of python scripts. The update process takes around 2 days, depending on the parallelization capacities. In terms of dependencies, MACADAM requires Python 3, the Pandas package and a valid license of Pathway Tools that have to be installed. All other dependencies are included by default in the python 3 setup. MACADAM can be updated at each RefSeq release (i.e. addition of new high-quality annotated genomes). MACADAM automatically downloads the new index summary from RefSeq. MACADAM then builds script downloads and processes all the genomes that matched our quality standards and launches Pathway Tools on each one of them. This is the crucial part in terms of computing power. In order to save time, the process is parallelized on the cluster of the GenoToul Bioinformatics Platform (http://bioinfo.genotoul.fr/) that provides access to high-performance computing resources. To do this, we process genomes by batches of 50 genomes and the whole step takes ~1 day. This step needs at least 8 GB of RAM for each batch. The generation of the unique ID and the calculation of the PS take around 6 h each due to file movements and extraction of archives. MACADAM can take up to 600 GB of disk space during the construction of the PGDBs, which is the most critical part. After compression, this file takes up 150 GB of disk space.

**Who:** MACADAM database benefits from the GenoToul Bioinformatics Platform facilities, including a permanent staff and technical support. The database will be available for downloading and querying as long as it demonstrates its utility for the research community. Moreover, the python script pipeline is available on the GitHub repository (GitHub URL: https://github.com/maloleboulch/MACADAM-Database).

**How often:** We intend to update MACADAM every 6 months so as to benefit from the latest information on genome annotation from RefSeq and prokaryotic classification from NCBI taxonomy.

## Querying the MACADAM database

### Input query

MACADAM provides users with metabolic pathways and functional annotations about taxonomic names. Thus, the user has to query MACADAM with one or several species and/or one or several taxonomic names (if several, they have to be separated by a comma). For more precise queries on taxonomic names, the taxonomic rank can be specified thanks to a drop-down menu (phylum, order, class, family, genus or species). Otherwise, the user has to query on ‘all ranks’. Thus, if the user specifies ‘coli’ and ‘genus’, *E. coli* will not be a match. One specificity of MACADAM is that if the queried organism/taxonomic name has no link to functional information, the user still obtains functional information about the upper rank of the target. To limit this, the ‘Strict taxonomy’ option allows the user to query only on target organism/taxonomic names and not beyond the specified taxonomic ranks. The search can also be refined by specifying (i) the full name (or part of the name) of a metabolic pathway or a functional feature (e.g. a query with ‘nitrate’ limits output information to 11 MetaCyc pathway names, 4 functional annotations from FAPROTAX and 1 functional annotation from the IJSEM phenotypic database), (ii) a MetaCyc class hierarchy ID (e.g. a query with ‘denitrification’ limits the output to 3 MetaCyc metabolic pathways and 4 FAPROTAX functional annotations), (iii) a specific PS (min score = 1 means that MACADAM returns only complete metabolic pathways), (iv) metabolite names (*n* = 7921), (v) reaction names (*n* = 592), (vi) enzyme names (*n* = 7440) and/or (vi) EC numbers (*n* = 2134). All search fields can be filled with complete or incomplete strings.

### Web interface: MACADAM explore

**Figure 5:**
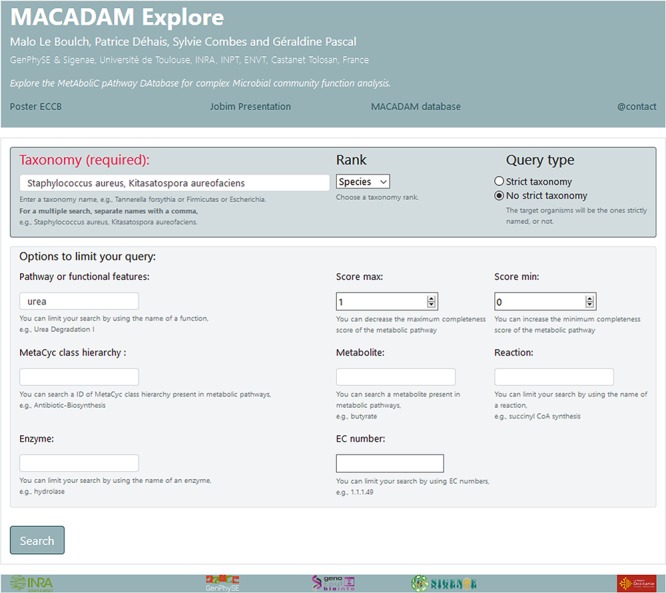
Screenshot of MACADAM Explore website showing the query of all functional information containing the word ‘urea’ in the species ‘*Staphylococcus aureus*’ and ‘*Kitasatospora aureofaciens*’.

We have created a web interface called MACADAM Explore (Figure 5) to facilitate the consultation of the MACADAM database. MACADAM Explore is built on an Apache Hypertext Transfer Protocol (HTTP) server. It provides commands to search and retrieve the data in the database. Python CGI is used for the front–end web interface. All common gateway interfaces and database interfacing scripts are written in the Python programming language. The database file (SQLite format) with a query script is downloadable at http://macadam.toulouse.inra.fr.

### Output file

**Figure 6:**
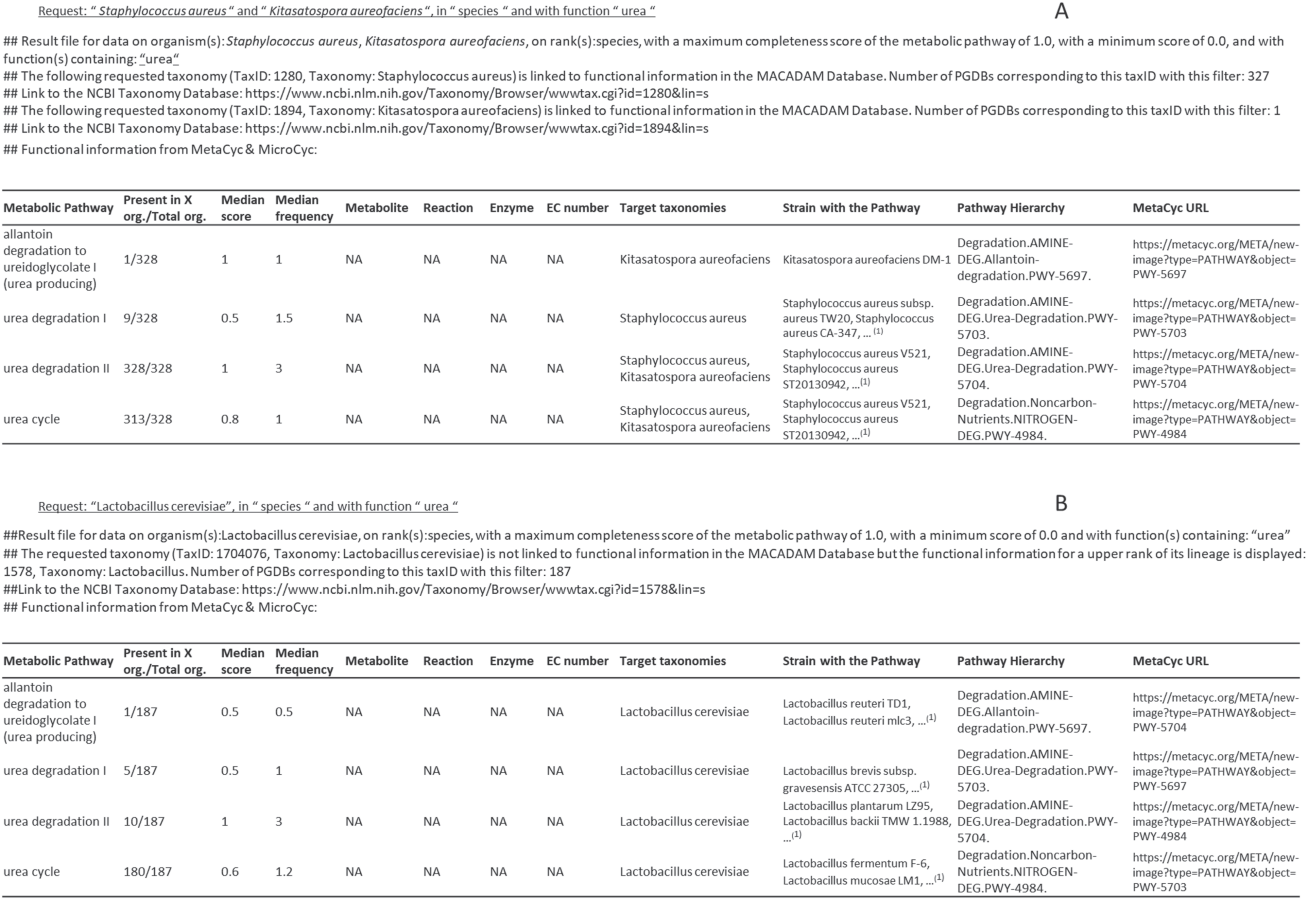
Examples of output files corresponding to requests on MACADAM. (**A**) The user has searched for all metabolic pathways in *S. aureus* and *K. aureofaciens*, using the term ‘urea’ in the function field text. (**B**) The user has searched for all metabolic pathways in *Lactobacillus cerevisiae* using the term ‘urea’ in the function field text. Since there is no data on this organism in MACADAM, the information was searched for higher up in the taxonomy hierarchy, i.e. *Lactobacillus*. ^(1)^List of organisms in MACADAM with the targeted metabolic pathway.

MACADAM returns a Tabular Separated Value (TSV) file as output (Figure 6) that is downloadable on a personal computer. This output contains a query reminder and details on the matching taxonomy in MACADAM. Moreover, it contains the list of functional information that responds to input criteria. The second column provides the number of organisms that have the targeted pathway. A high proportion shows a high presence of targeted pathways among targeted taxonomic names. For example, as shown in Figure 6A, the ‘Urea Degradation II’ pathway is present in all 328 genomes that match taxID 1280 and 1894 corresponding to *Staphylococcus aureus* and *Kitasatospora aureofaciens,* although the ‘Urea Degradation I’ pathway is present in only nine genomes of *S. aureus,* and the ‘allantoin degradation to ureidoglycolate’ is only present in one strain of *K. aureofaciens*. The output also provides information about the median value of the PS and PFS. There are also columns corresponding to metabolite names, reaction names, enzyme names and EC numbers entered as filtered input by the user. The last columns contain targeted taxonomy names, the list of strains that have the pathways and the MetaCyc metabolic pathway hierarchy with the corresponding URL.

## Utility and discussion

### 

#### MACADAM is designed to collect bacterial and archaeal genomes of the highest quality associated with the highest quality annotations

Reliable data are needed to infer the functional potential of complex prokaryotic communities. Indeed, obsolete functional information can lead to inaccurate insights (36). As far as possible, MACADAM avoids sequentially spurious annotations. In the MACADAM database, reliability is ensured by filters on genome quality, meaning that only complete genomes have been taken from RefSeq. Moreover, to ensure up-to-date annotations, we compute PGDBs with Pathway Tools at each release, based on RefSeq, NCBI taxonomy, MetaCyc, MicroCyc, FAPROTAX and the IJSEM phenotypic database.

**Figure 7:**
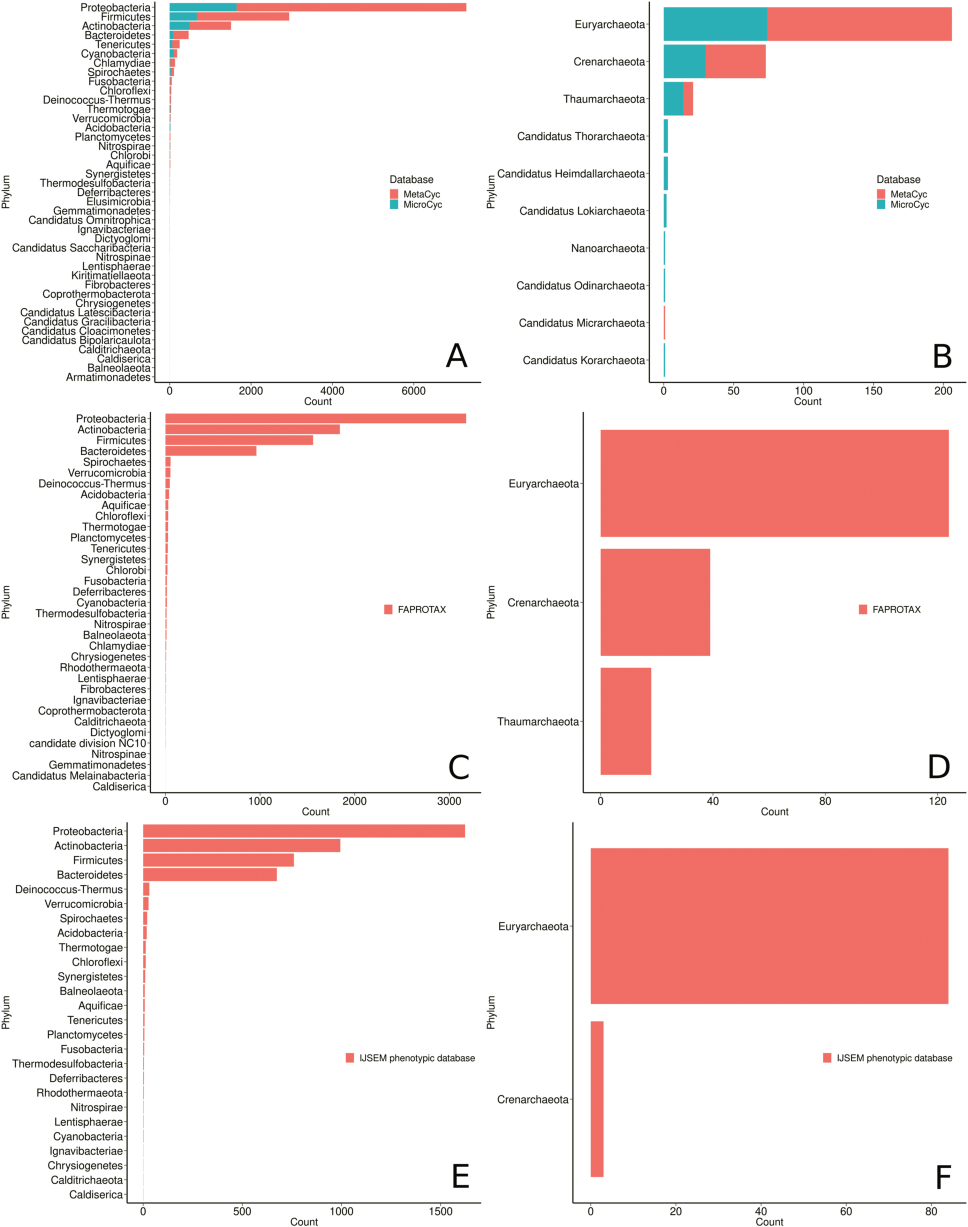
Phyla distribution in the MACADAM database according to their database of origin. (**A**) MetaCyc and MicroCyc for bacterial organisms, (**B**) MetaCyc and MicroCyc for archaea organisms, (**C**) FAPROTAX for bacterial organisms, (**D**) FAPROTAX for archaea organisms, (**E**) IJSEM phenotypic database for bacterial organisms and (**F**) IJSEM phenotypic database for archaea organisms.

#### MACADAM PGDBs cover all phyla recognized by the List of Prokaryotic names with Standing in Nomenclature

**Figure 8:**
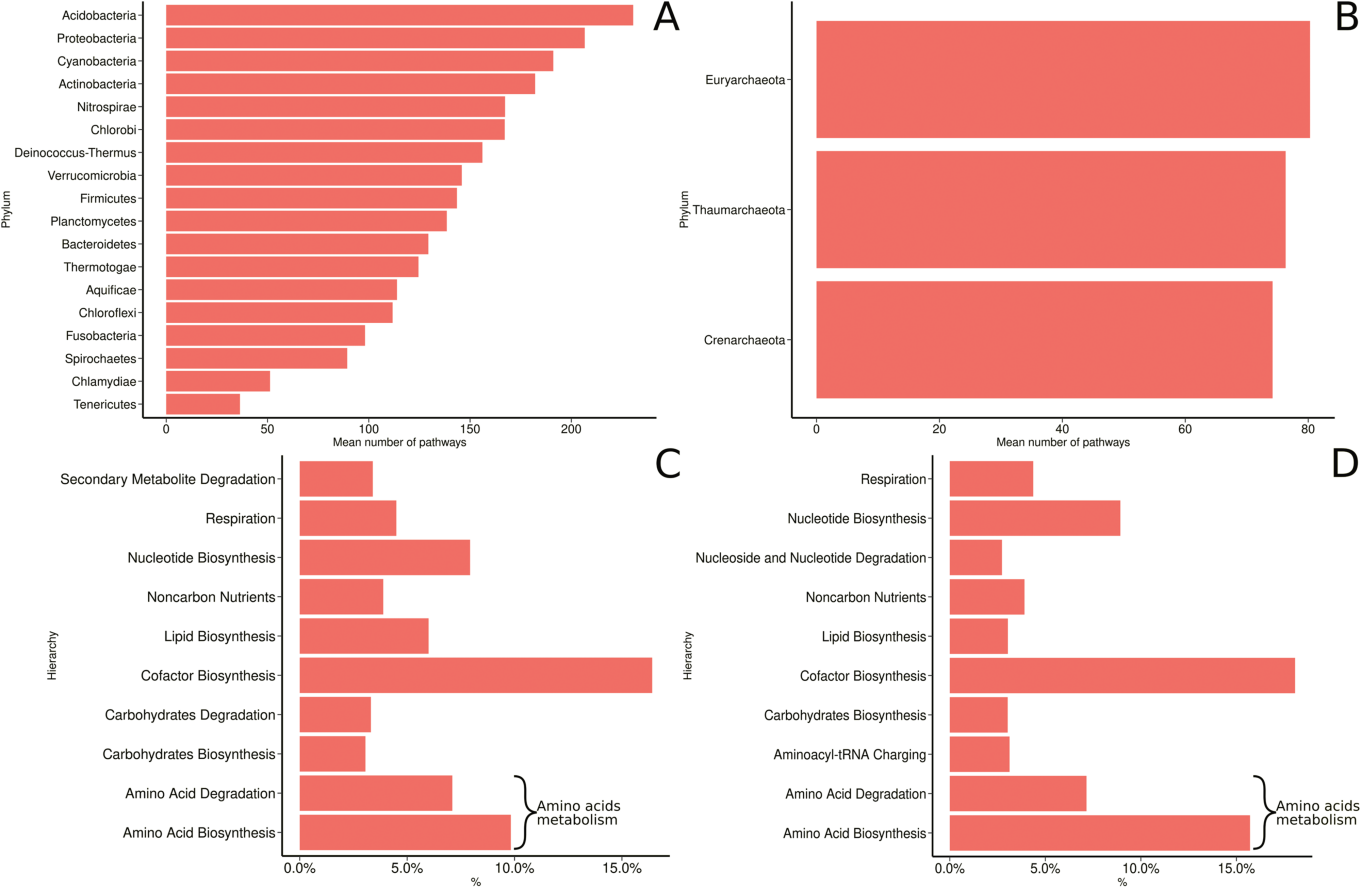
MACADAM functional diversity for phyla with >10 PGDBs in MACADA (**A**) among bacterial phyla, (**B**) among archaea phyla, (**C**) the 10 main hierarchical groups of pathways in all bacterial organisms and (**D**) the 10 main hierarchical groups of pathways in all archaea organisms.

 (LPSN; http://www.bacterio.net/) and the 10 other newly proposed phyla from the NCBI taxonomy (Figure 7). Proteobacteria is the most prevalent phylum and accounts for >55% of the genomes collected in MACADAM, followed by the *Firmicutes* and *Actinobacteria* phyla that account for >22% and 11% of the collected genomes, respectively. This pre-eminence is probably explained by the research effort devoted to these phyla by biologists. Accordingly, *Escherichia* and *Salmonella* are the prevalent genera in the database (5.1% and 4.3% of the database, respectively). Interestingly, according to Figure 7, the *Acidobacteria* phylum is weakly represented in MACADAM (only 21 PGDBs, 19 of which are from MicroCyc). But these PGDBs include the highest number of metabolic pathways (mean = 230; min = 138; max = 298; Figure 8). Acidobacteria is one of the most widespread phyla, but few organisms of this phylum are cultivated and sequenced (37) and may explain the low representation of available high-quality genome that could be included in MACADAM. The high number of metabolic pathway present in these widespread phyla may indicate that, despite few sequenced organisms, these are subject to particular care in terms of functional annotation. In parallel, we should also recall that the most common metabolic pathways in MACADAM belong to cofactor biosynthesis (bacteria: 16.4%; archaea: 18.06%) and the metabolism of amino acids (bacteria: 17.7%; archaea: 22.86%).

**Table 4:** Statistics on the L-lysine fermentation to acetate and butanoate pathway (MetaCyc ID is P163-PWY; values in brackets are minimum and maximum values

**MetaCyc ID: P163-PWY characteristics**	
Number of reactions in the complete pathway^*^	10
Number of bacteria in which this pathway is present	756
Median number of unique enzymes present in this pathway in organisms	4 [1–10]
Median number of enzymes present in this pathway in organisms	7 [1–70]
Key reaction^*^	1 (enzyme classification: 5.4.3.2)
PS	0.4 [0.1–1]
PFS	0.7 [0.1–7]

^*^Value found in a MetaCyc flat file; a key reaction is a reaction that is specific to a single pathway, i.e. a reaction that is not found in any other pathway.

#### MACADAM allows users to refine queries using PSs

PSs range from 0 to 1 (Table 2). A value of 1 indicates that all the enzymes required for the pathway are present in the genome of the organism. A value of 0 indicates that none of the enzymes are present in the targeted genome. In the latter case, the pathway is still present because of the functional inference applied by MetaCyc experts, information that they cross-reference with phenotypic evidence described in the literature. MACADAM contains eight pathways in 1519 organisms with a PS equal to 0. The PFS ranges from 0 to 77 (Table 2). *Nocardia nova SH22a* has the maximum PFS for its long-chain fatty acid activation pathway. This pathway comprises only one reaction and is part of a longer lipid biosynthesis pathway.

Table 4 is an example of statistics performed on L-lysine fermentation to acetate and butanoate pathway output (MetaCyc ID: P163-PWY), a pathway of interest in understanding the interactions between host and gut microbiota in health and disease. In fact, butyrate is a microbial fermentation product that is used as an energy source by enterocytes and whose signaling properties are involved in multiple functions of enterocytes, including cell differentiation, gut tissue development, immune modulation, oxidative stress reduction and diarrhea control (38). Ten enzymes are involved in the L-lysine fermentation to acetate and butanoate pathway. The pathway is present in 992 different organisms in MACADAM. This complete pathway is composed of 10 enzymes. If the median value of the PS is equal to 0.4, this means that four reactions have at least one annotated enzyme in the pathway. If the median value of the PFS is equal to 0.7, this means these 4 reactions have >1 associated enzymes, 7 enzymes for 10 reactions in all (Figure 2). According to MetaCyc, the 5.4.3.2 reaction is a key reaction in this pathway. Therefore, if the organism contains this reaction, the whole pathway will be identified for the organism. These PS and PFS data are useful to biologists for data mining.

An important feature of MACADAM is its ability to **infer functional annotations for taxa with no associated genomic sequences using the taxonomy**. In the case of a taxon with no functional information in MACADAM, i.e. missing in MetaCyc, MicroCyc, FAPROTAX and the IJSEM phenotypic database, we provide information for the upper taxonomy rank. For example, if a species has no functional information, MACADAM automatically requests functional information at the genus level, just like FAPROTAX. However, MACADAM can do this at any taxonomic rank, while FAPROTAX is limited to the order rank. In MACADAM, in this case, all annotations for organisms belonging to this genus are shown in the output file. As for FAPROTAX, it indicates functional information described in the literature at the genus rank if all described species of the genus have been shown to exhibit the given functional information and not the addition of functional information about all of the organisms belonging to this rank. Thanks to the column that gives the number of organisms with the targeted pathway (column 2 in Figure 6B), it is possible to see pathways that are more or less conserved in the taxon of interest. For example, ‘urea cycle’ is a pathway conserved in most of *Lactobacillus*, unlike the ‘urea degradation I or II’ pathways. Thus, this feature provides functional information about organisms with no functional information based on related taxonomic species.

## Conclusions

MACADAM was designed for the microbiology community as a functional annotation information database based on multiple sources of data on functional annotations and on metabolic pathways (MetaCyc, MicroCyc, FAPROTAX and the IJSEM phenotypic database). The database is also based on the complete and interoperable NCBI taxonomy. MACADAM covers all known bacterial and archaeal phyla, as of February 2019. A standardized score enables quick comparison and comprehension of the potential presence of a pathway. If there is no functional information on the taxonomy entered, MACADAM automatically checks the upper taxonomic rank in order to provide functional information associated with related organisms to users. MACADAM can be explored via metabolites, reactions, enzymes, EC numbers or specific pathways. A user-friendly web interface makes querying easy. MACADAM will be useful to all biologists who need to determine the functional potential of a prokaryotic species or any other taxonomic rank. Since the source code to build MACADAM is available to everyone (GitHub URL: https://github.com/maloleboulch/MACADAM-Database), MACADAM can be included in any functional inference tool able to integrate the abundance tables of complete microbial communities generated, among others, we plan to include MACADAM in the FROGS software (39) to analyze amplicon metagenomics data.

## Notes

**Github URL:** https://github.com/maloleboulch/MACADAM-Database

**Database URL:** macadam.toulouse.inra.fr
